# Variation in adverse drug events of opioids in the United States

**DOI:** 10.3389/fphar.2023.1163976

**Published:** 2023-03-24

**Authors:** Edward Y. Liu, Kenneth L. McCall, Brian J. Piper

**Affiliations:** ^1^ Department of Medical Education, Geisinger Commonwealth School of Medicine, Scranton, PA, United States; ^2^ Department of Pharmacy Practice, Binghamton University, Binghamton, NY, United States; ^3^ Center for Pharmacy Innovation and Outcomes, Geisinger, Danville, PA, United States

**Keywords:** opiate, oxycodone, hydrocodone, fentanyl, meperidine

## Abstract

**Background:** The United States (US) ranks high, nationally, in opioid consumption. The ongoing increase in the misuse and mortality amid the opioid epidemic has been contributing to its rising cost. The worsening health and economic impact of opioid use disorder in the US warrants further attention. We, therefore, assessed commonly prescribed opioids to determine the opioids that were over-represented versus under-represented for adverse drug events (ADEs) to better understand their distribution patterns using the Food and Drug Administration’s Adverse Event Reporting System (FAERS) while correcting for distribution using the Drug Enforcement Administration’s Automation of Reports and Consolidated Orders System (ARCOS). Comparing the ratio of the percentage of adverse drug events as reported by the FAERS relative to the percentage of distribution as reported by the ARCOS database is a novel approach to evaluate post-marketing safety surveillance and may inform healthcare policies and providers to better regulate the use of these opioids.

**Methods:** We analyzed the adverse events for 11 prescription opioids, when correcting for distribution, and their ratios for three periods, 2006–2010, 2011–2016, and 2017–2021, in the US. The opioids include buprenorphine, codeine, fentanyl, hydrocodone, hydromorphone, meperidine, methadone, morphine, oxycodone, oxymorphone, and tapentadol. Oral morphine milligram equivalents (MMEs) were calculated by conversions relative to morphine. The relative ADEs of the selected opioids, opioid distributions, and ADEs relative to distribution ratios were analyzed for the 11 opioids.

**Results:** Oxycodone, fentanyl, and morphine accounted for over half of the total number of ADEs (*n* = 667,969), while meperidine accounted for less than 1%. Opioid distributions were relatively constant over time, with methadone repeatedly accounting for the largest proportions. Many ADE-to-opioid distribution ratios increased over time, with meperidine (60.6), oxymorphone (11.1), tapentadol (10.3), and hydromorphone (7.9) being the most over-represented for ADEs in the most recent period. Methadone was under-represented (<0.20) in all the three periods.

**Conclusion:** The use of the FAERS with the ARCOS provides insights into dynamic changes in ADEs of the selected opioids in the US. There is further need to monitor and address the ADEs of these drugs.

## Introduction

Opioids have been commonly prescribed to treat moderate to severe pain for various conditions, including cancer and trauma. Fentanyl, methadone, and oxycodone are examples of commonly prescribed opioids. Overuse of these drugs can lead to adverse drug events (ADEs), tolerance, dependence, addiction, overdose, and death. Drug overdose deaths increased four-fold from 1999 to 2017, with opioid-related deaths accounting for about two-thirds of the deaths (Singh et al., 2019). The Centers for Disease Control (CDC) has recently indicated that the number of drug overdose deaths increased by nearly 5% from 2018 to 2019, with over 70% of the 70,630 drug-related deaths in 2019 involving opioids (Centers for Disease Control and Prevention, 2021). Although the volume of opioids prescribed in the US decreased from 2010 to 2015 after peaking in 2011, the amount is still significantly higher relative to 1999 (Guy, 2017; Mack et al., 2018; Piper et al., 2018). An analysis of the International Narcotics Control Board records from 2015 to 2017 revealed that 10% of the world’s population consumed 89% of the world’s supply of prescription opioids. Furthermore, the US ranked third for the highest opioid consumption *per capita* (Richards et al., 2022). The fatalities and overdoses from the misuse of these analgesics were responsible for $1.02 trillion in costs in the US in 2017 (Florence et al., 2021). The detrimental health and economic impact of both pain and opioid use disorder treatments in the US warrants further attention.

A recent report examining the national patterns in opioid exposure reported to the US poison control centers indicated that the proportion of exposure with adverse drug events (ADEs) increased despite the overall decrease in the frequency and rate of opioid exposure from 2011 to 2018 (Rege et al., 2021). ADEs are reported in the US Food and Drug Administration Adverse Event Reporting System (FAERS), a large government database that consists of ADEs and medication error reports submitted through the MedWatch program primarily from healthcare professionals (Zhou and Hultgren, 2020). In addition to using the FAERS database to quantify the adverse effects, we used the Drug Enforcement Administration’s (DEA) Automation of Reports and Consolidated Orders System (ARCOS), a comprehensive data collection system, where schedule II and III controlled substances are mandatorily reported when distributed to pharmacies, hospitals, narcotic treatment programs (NTPs), and long-term care facilities (Piper et al., 2018; U.S. Drug Enforcement Administration, 2022). We used both databases to identify the ADEs of several common schedule II and III prescription opioids relative to their distribution in the US for the past one and a half decades. This analysis identifies the opioids that were over- or under-represented for ADEs relative to their use.

## Methods

### Procedures

FDA FAERS and ARCOS databases were queried from 2006 to 2021 to examine the ADEs and distribution of the 11 opioids. These opioids were selected based on previous studies and their status as being FDA-approved and commonly prescribed. Nine of them are used primarily for pain, namely, codeine, fentanyl, hydrocodone, hydromorphone, meperidine, methadone, morphine, oxycodone, oxymorphone, and tapentadol, and two of them are mainly used for opioid use disorders (OUD), buprenorphine and methadone (Modarai et al., 2013; Mack et al., 2018; Cabrera et al., 2019; Singh et al., 2019; Veronin et al., 2019; Eidbo et al., 2022). We separated the analysis into three time periods based on pre- (2006–2010), intra- (2011–2016), and post-peak (2017–2021) opioid distribution time intervals. Specifically, 2011 was the peak year of opioid count by morphine milligram equivalents (MMEs) (Piper et al., 2018). The search involved both generic and brand opioid names indicated in the FAERS database with ADEs including misuse, overdoses, serious cases, and deaths (U.S. Food and Drug Administration, 2022). Supplementary Table S1 indicates the search terms used for these opioids. Additionally, the DEA ARCOS database is comprehensive and has input from pharmacies, hospitals, distributors, and wholesalers regarding schedule II and III controlled substances in the US. It includes controlled substances for medical use and is, therefore, a very inclusive and valid database (Bokhari et al., 2005). Analyses of oxycodone from the ARCOS showed a high correlation (r = .985) with a state prescription drug monitoring program (Piper et al., 2018). The procedures were approved as exempted by the IRB of Geisinger and the University of New England.

### Statistical analyses

The total oral MME was calculated based on the weight of all 11 opioids and expressed in three periods (2006–2010; 2011–2016; 2017–2021) for the US, excluding the US territories. Hereafter, these periods are referred to as the first, second, and third, respectively. The first period showed increases in prescription opioid distribution, 2011 was the peak year, and the third period showed a further decline in the opioids used for pain and an escalation in OUD treatment (Piper et al., 2018; Collins et al., 2019; Azar et al., 2020). The top three reaction groups and reactions were reported for each opioid from 2006–2021 with percentages indicating the amount relative to the total number of adverse events within that period. Three analyses were also completed for each period: (1) the frequency of ADEs of each opioid based on the FAERS, (2) the percentage of total opioid distribution based on the ARCOS, and (3) FAERS to ARCOS ratios. The oral MME was calculated to correct for the relative potency of each opioid relative to morphine. The conversions were as follows: buprenorphine (10), codeine (0.15), fentanyl base (75), hydrocodone (1), hydromorphone (4), meperidine (0.1), methadone (10), morphine (1), oxycodone (1.5), oxymorphone (3), and tapentadol (0.4) (Piper et al., 2018; Eidbo et al., 2022). For buprenorphine, the CDC MME conversion charts ceased to include the opioid in 2016, while at a low dose, buprenorphine can produce significantly greater opioid responses than morphine. Although morphine (a full agonist with low potency) response is dose-related until it reaches 100% maximal response, buprenorphine (partial agonist) effects reach the peak, at which point, further increases in doses within the clinical range do not increase the magnitude of the response. This concept of potency is important for understanding why buprenorphine should not be converted to MMEs for purposes of assessing the overdose risk based on the daily opioid dose; according to the American Society of Addiction Medicine, “Opioid dosing guidelines developed for chronic pain, expressed in morphine milligram equivalents (MMEs), are not applicable to medications for the treatment of opioid use disorders.” Therefore, the authors selected a conversion factor, for buprenorphine to morphine, of 10 from a range of values documented in potency studies for the purpose of this pharmacoepidemiologic study (ASAM, 2020). Methadone’s MME was calculated based on the dose (Centers for Disease Control and Prevention, 2023). We decided to conduct an average of narcotic treatment programs (12) and other sources (8) for an MME of 10. Additionally, there is a range of the equianalgesic dose ratio of methadone established from previous studies with a median dose ratio ranging 5.98–16.27 (Lawlor et al., 1998) relative to morphine and 0.81–2.47 for hydromorphone (Ripamonti, et al., 1998). As for fentanyl, 75 was selected as it is 50–100 times more potent than morphine (Higashikawa and Suzuki, 2008; Volpe et al., 2011).

We identified any ratio >1.0 as an over-representation and <1.0 as under-representation of the ADEs of the opioid when correcting for distribution, for example, an opioid which accounted for 10% of ADEs but 5% of the distribution would have a ratio of 2.0 (i.e., over-represented). We extracted the top three reaction groups and reactions to outline the common adverse drug effects associated with the selected opioids, indicating which adverse effects may have been contributing to the reports. We chose the top three reports as they made up >60–75% of the ADEs. It is important to note that the death report percentages are overestimated as large public databases involve individuals who can submit more than one report (Stephenson and Hauben, 2007; United States Drug Enforcement Administration, 2016). We also prepared a Supplementary Table S1 that differentiated the death reports either by “outcome” or “reaction” for the opioids, meaning that not all reports are associated with direct deaths from the drugs. Data analysis and figure preparation were completed with GraphPad Prism, version 9.3.1.

## Results

We queried data from FAERS and ARCOS databases for the 11 opioids from 2006 to 2021. Supplementary Figure S1 indicates the percentage of ADE reports submitted by healthcare professionals to the FAERS for the 11 opioids from 2006 to 2021. Almost one-third (31.2%) of the reports were from providers. Codeine (72.9%), meperidine (70.5%), and methadone (68.1%) had the most submissions from healthcare workers, while the remaining eight opioid ADEs were submitted mainly from patients. Table 1 indicates the ADE reports from 2006 to 2021 obtained from the FAERS. Oxycodone, fentanyl, and morphine were responsible for over half (55.2%) of the total number of ADEs (*n* = 667,969), while meperidine accounted for less than 1% of them. The top three most common reaction groups included injury, poisoning, and procedural complications; general disorders and administration site conditions; and psychiatric disorders. The common specific reactions consisted of abnormal drug effects (e.g., dependence, hypersensitivity, and ineffectiveness), overdose, and death. The death rates varied among the opioids, with oxymorphone having the largest proportion of death as the “reaction” (40.6%) and death as an “outcome” (70.6%), while meperidine had the least with 1.4% and 7.4% as the reaction and outcome, respectively (Supplementary Table S2).

**TABLE 1:** Adverse event reports by the count and percentage in the US Food and Drug Administration’s Adverse Effect Reporting System for 11 prescription opioids for the 2006–2021 period. The three most common reaction groups and reactions are shown.

Opioid	Reaction group	Reaction
Oxycodone (159,441); 23.9%	1. Psychiatric disorders; *n* = 99,132 (62.2%)	1. Drug dependence; *n* = 74,721 (46.9%)
	2. General disorders and administration site conditions; *n* = 79,824 (50.1%)	2. Overdose; *n* = 38,543 (24.2%)
	3. Injury, poisoning, and procedural complications; *n* = 75,491 (47.3%)	3. Pain; *n* = 27,545 (17.3%)
Fentanyl (106,644); 16.0%	1. Injury, poisoning, and procedural complications; *n* = 56,480 (53.0%)	1. Death; *n* = 16,309 (15.3%)
	2. General disorders and administration site conditions; *n* = 51,784 (48.6%)	2. Toxicity to various agents; *n* = 15,225 (14.3%)
	3. Psychiatric disorders; *n* = 23,740 (22.2%)	3. Overdose; *n* = 11,200 (10.5%)
Morphine (102,411); 15.3%	1. General disorders and administration site conditions; *n* = 47,624 (46.5%)	1. Drug dependence; *n* = 28,830 (28.2%)
	2. Injury, poisoning, and procedural complications; *n* = 47,310 (46.2%)	2. Overdose; *n* = 20,224 (19.7%)
	3. Psychiatric disorders; *n* = 43,773 (42.7%)	3. Death; *n* = 17,088 (16.7%)
Buprenorphine (80,685); 12.1%	1. General disorders and administration site conditions; *n* = 47,311 (58.6%)	1. Drug dependence; *n* = 13,011 (16.1%)
	2. Injury, poisoning, and procedural complications; *n* = 34,058 (42.2%)	2. Death; *n* = 12,634 (15.7%)
	3. Psychiatric disorders; *n* = 20,589 (24.5%)	3. Overdose; *n* = 10,981 (13.6%)
Hydromorphone (64,454); 9.6%	1. Injury, poisoning, and procedural complications; *n* = 37,081 (57.5%)	1. Drug dependence; *n* = 25,321 (39.3%)
	2. General disorders and administration site conditions; *n* = 35,763 (55.5%)	2. Overdose; *n* = 18,430 (28.6%)
	3. Psychiatric disorders; *n* = 31,762 (49.3%)	3. Death; *n* = 15,064; (23.4%)
Hydrocodone (44,204); 6.6%	1. Injury, poisoning, and procedural complications; *n* = 24,986 (56.5%)	1. Death; *n* = 12,990 (29.4%)
	2. General disorders and administration site conditions; *n* = 24,626 (55.7%)	2. Drug dependence; *n* = 10,894 (24.6%)
	3. Psychiatric disorders; *n* = 15,673 (35.5%)	3. Toxicity to various agents; *n* = 10,829 (24.5%)
Oxymorphone (31,154); 4.7%	1. Injury, poisoning, and procedural complications; *n* = 20,013 (64.2%)	1. Death; *n* = 12,654 (40.6%)
	2. General disorders and administration site conditions; *n* = 17,132 (55.0%)	2. Toxicity to various agents; *n* = 9,757 (31.3%)
	3. Psychiatric disorders; *n* = 6,396 (20.5%)	3. Overdose; *n* = 7,160 (23.0%)
Tapentadol (29,290); 4.4%	1. Injury, poisoning, and procedural complications; *n* = 17,678 (60.4%)	1. Death; *n* = 11,579 (39.5%)
	2. General disorders and administration site conditions; *n* = 15,316 (52.3%)	2. Toxicity to various agents; *n* = 9,411 (32.1%)
	3. Psychiatric disorders; *n* = 4,188 (14.3%)	3. Overdose; *n* = 5,815 (19.9%)
Methadone (27,454); 4.1%	1. Injury, poisoning, and procedural complications; *n* = 14,405 (52.5%)	1. Toxicity to various agents; *n* = 5,226 (19.0%)
	2. Psychiatric disorders; *n* = 12,229 (51.9%)	2. Drug dependence; *n* = 4,339 (15.8%)
	3. General disorders and administration site conditions; *n* = 11,155 (40.6%	3. Drug abuse; *n* = 3,937 (14.3%)
Codeine (16,731); 2.5%	1. Immune system disorders; *n* = 7,075 (42.3%)	1. Drug hypersensitivity; *n* = 6,576 (39.3%)
	2. Injury, poisoning, and procedural complications; *n* = 5,702 (34.1%)	2. Toxicity to various agents; *n* = 2,538 (15.2%)
	3. General disorders; *n* = 4,611 (27.6%)	3. Drug ineffective; *n* = 1,210 (7.2%)
Meperidine (5,501); 0.82%	1. Immune system disorders; *n* = 3,143 (57.1%)	1. Drug hypersensitivity; *n* = 2,920 (53.1%)
	2. General disorders and administration site conditions; *n* = 1,598 (29.0%)	2. Drug ineffective; *n* = 433 (7.9%)
	3. Injury, poisoning, and procedural complications; *n* = 1,096 (19.9%)	3. Pain; *n* = 350 (6.4%)

**FIGURE 1:**
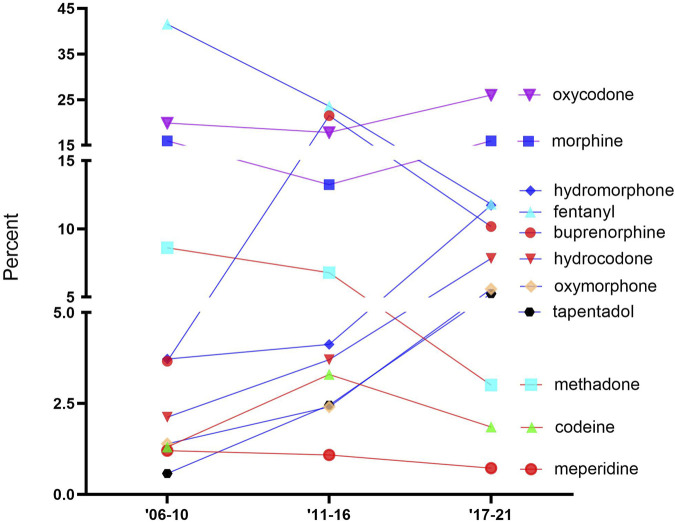
Percentage of adverse drugs events (ADEs) of the 11 prescription opioids obtained from the FDA Adverse Event Reporting System over time.

Figure 1 shows the percentage of ADEs for each opioid. The opioids were classified into three groups in 2017–2021, namely, high (>15%): oxycodone and morphine; intermediate (5%–15%): hydromorphone, fentanyl, buprenorphine, hydrocodone, oxymorphone, and tapentadol; and low (<5%): methadone, codeine, and meperidine. Oxycodone was consistently high across all periods: 2006–2010 (19.9%), 2011–2016 (17.8%), and 2017–2021 (26.0%). Fentanyl accounted for the largest portion of ADEs in the first two periods (2006–2010 (41.6%) and 2011–2016 (23.6%)) but decreased greatly since the second period (−49.9%). Methadone showed a noticeable decrease (−55.8%) from the second to the third period, while oxymorphone indicated a marginal (+114.5%) increase. Codeine and meperidine accounted for less than 5% of the total ADE reports in all periods.

**FIGURE 2:**
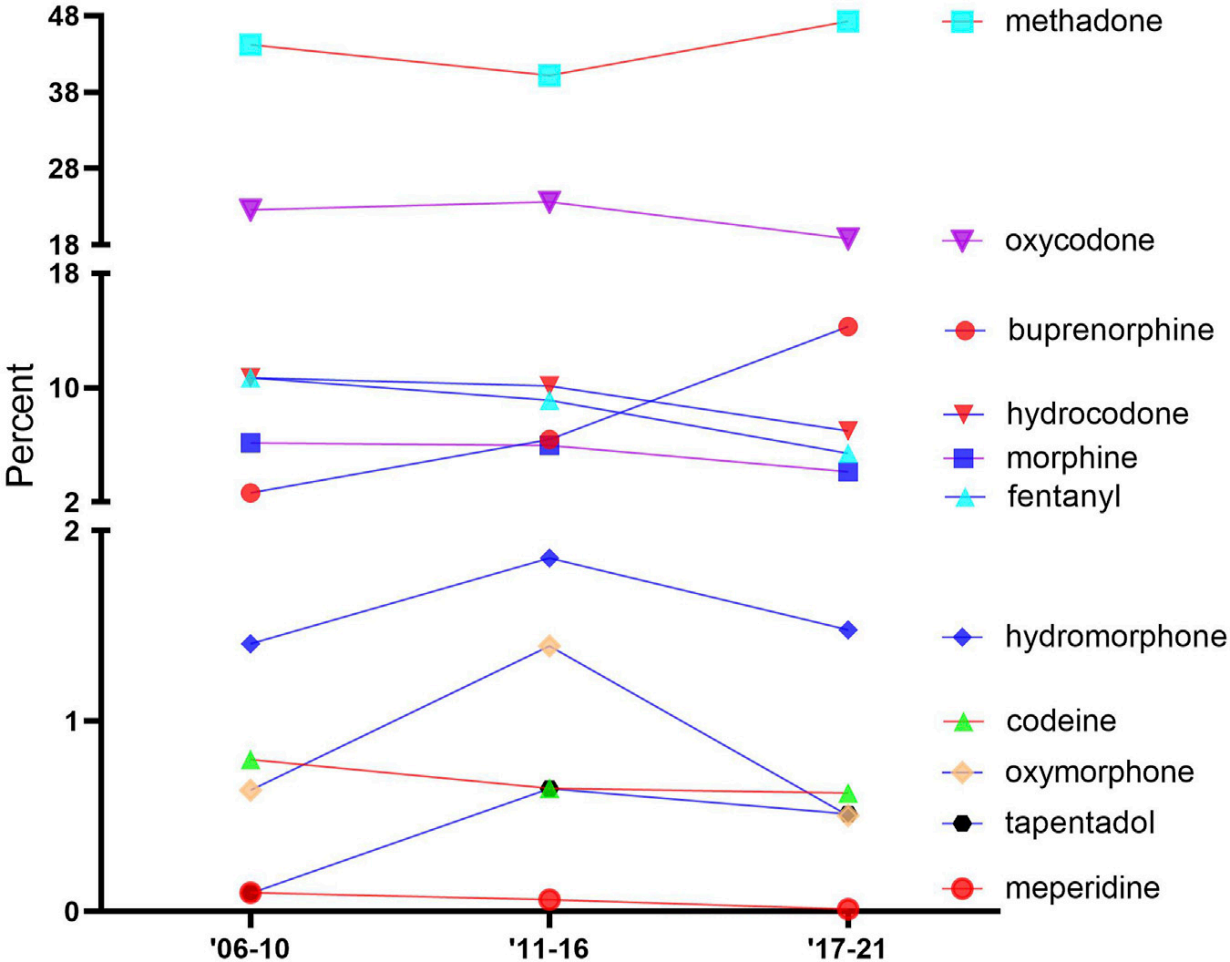
Percentage of the total morphine mg equivalent (MME) of the distribution of 11 prescription opioids, as reported by the Drug Enforcement Administration’s Automated Reports and Consolidated Orders System (ARCOS) over time.

Figure 2 shows the percentage of the total MMEs due to each opioid over time. The opioids were classified into three groups, which were generally stable over time, namely, high: methadone and oxycodone; intermediate: buprenorphine, hydrocodone, morphine, and fentanyl; and low: hydromorphone, codeine, oxymorphone, tapentadol, and meperidine. Methadone accounted for over two-fifths of the total distribution: 2006–2010 (44.2%), 2011–2016 (40.2%), and 2017–2021 (47.3%). Codeine, tapentadol, and meperidine consistently made up less than 1% of the distribution.

**FIGURE 3:**
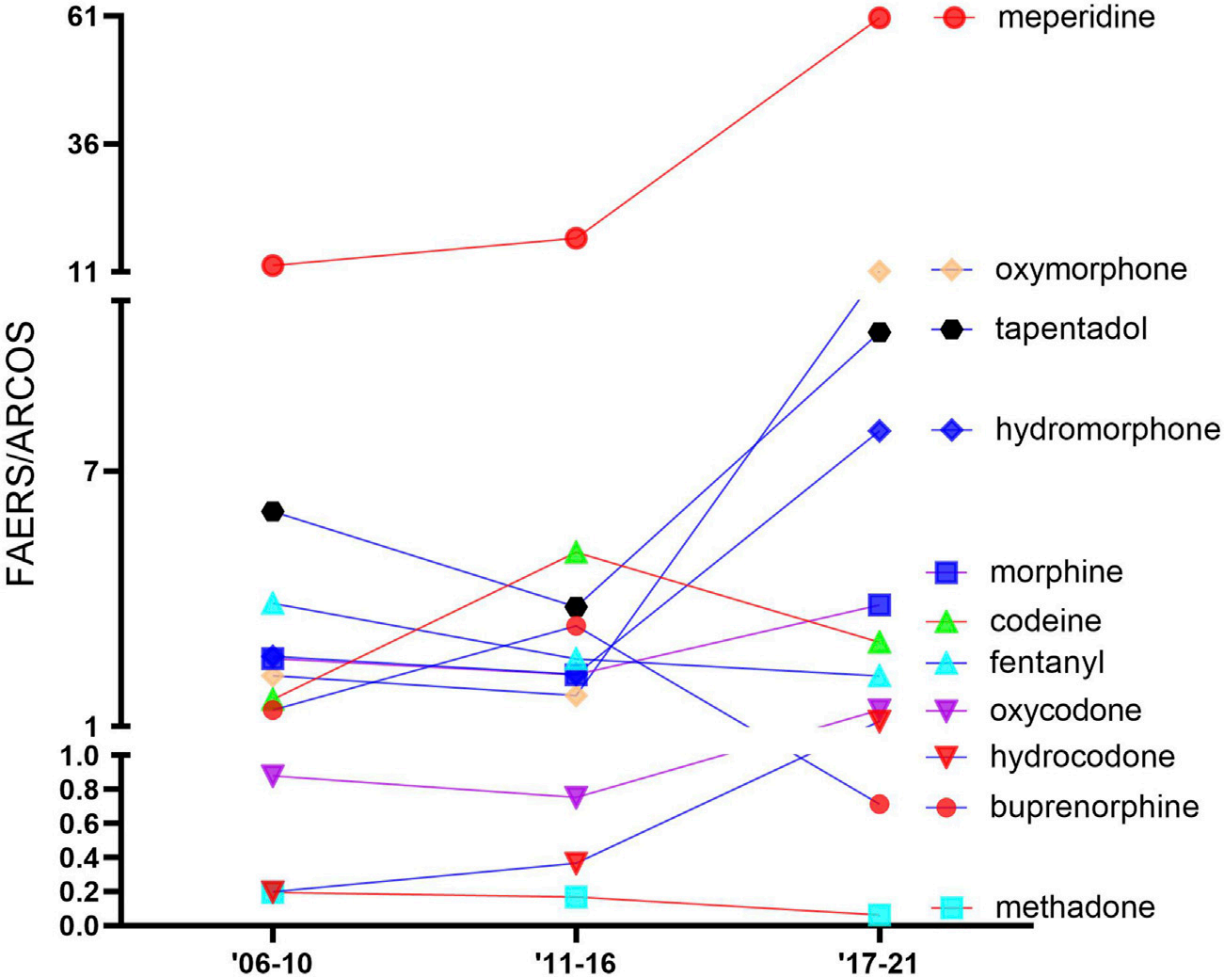
Ratio of the percentage of adverse drug events, as reported by the US Food and Drug Administration’s Adverse Events Reporting System relative to the percent of distribution as reported by the Drug Enforcement Administration’s Automated Reports and Consolidated Orders System database for the 11 prescription opioids over time. Values greater than 1.0 are over-represented, and values less than 1.0 are under-represented.

Figure 3 shows the ADE-to-distribution ratio for each opioid for each period. Most opioids showed an over-representation in the FAERS-to-ARCOS ratio. The general pattern was of increases over time, with the most over-represented opioid in the third period being meperidine (60.6), followed by oxymorphone (11.1), tapentadol (10.3), and hydromorphone (7.9). Oxymorphone showed the largest increase (+542.2%) in its ratio from the second to the third period, followed by hydromorphone (+257.7%) and meperidine (+245.2%). Buprenorphine had the greatest decrease (−371.3%), followed by codeine (−71.0%) and fentanyl (−18.2%). Methadone was under-represented (<.20) in the three periods.

## Discussion

Our study identified the varied ADEs for 11 commonly used opioids. This is also the first report to describe the ADEs while correcting for the prevalence of each opioid’s US distribution.

Stronger opioids, like fentanyl (MME = 75), were associated with more frequent adverse events, while other opioids, like hydromorphone (MME = 4) and oxymorphone (MME = 3), had low adverse events relative to their distribution. Oxycodone, fentanyl, and morphine accounted for over half (55.2%) of the total number of ADEs (*n* = 667,969) with meperidine comprising less than 1% of it. Oxycodone shows high potential for misuse due to its high reinforcing characteristics and its administration methods, including pill crushing for immediate release and through IV injections, leading to high dependence (Kibaly et al., 2021; Table 1). Fentanyl with its high potency and abuse potential, as well as the tendency to be mixed with other drugs, may contribute to high ADEs across all three periods (United States Drug Enforcement Administration, 2018; Comer and Cahill, 2019; National Institute on Drug Abuse, 2021). Like other opioids, morphine tolerance can develop secondary to its continuous usage due to the changes in the receptor density and G-protein-coupled receptors and its signal transduction pathway (Listos et al., 2019). Although meperidine had a consistently low (<1%) distribution, its elevated ADE-to-distribution ratio may be because of its ability to cause lethal ADEs, including serotonin syndrome and psychological or physical dependence (Boyle et al., 2021). The distribution of this ubiquitous agent has continued to decline (Harrison et al., 2022).

Methadone and buprenorphine both showed increases in distribution in the past decade mostly due to their use for OUD, with buprenorphine also showing a 122.5% increase in hospital distribution in the past decade (Bishop-Freeman et al., 2021; Cicero et al., 2014; Eidbo et al., 2022; Furst et al., 2022; Mattick et al., 2014; Pashmineh Azar et al., 2020). These opioids have been commonly used to treat opioid dependence, and with an expanded Medicaid coverage, their prevalence has been rising (Mattick et al., 2014; Burns et al., 2016). The high distribution of oxycodone may be attributed to its common use and effectiveness for treating moderate-to-severe acute pain (Moradi et al., 2012; Davis and Liberman, 2021).

Given that meperidine demonstrated the lowest frequency of ADEs, it was surprising to find that its adverse effects were the most overly represented compared to its distribution (60.6), particularly in the third period (60.6). In contrast to Veronin et al. (2019), who found that oxycodone had high death-to-count percentages compared to other opioids, our report indicated oxycodone’s percentages hovered around 1% of the ratios throughout the study, as seen in Figure 3. The decline in oxycodone overdoses might be attributed to the reduction in abuse since the development of its extended release in late 2010 (Johnson et al., 2014).

Oxymorphone’s notable increase in adverse effects (+542.2%) relative to its distribution was unsurprising as it constantly had low counts throughout the three periods relative to other opioids. Oxymorphone as a schedule II drug has a high potency and misuse potential related to its euphoric effects explaining the huge increase in proportion, which might also explain its high proportion of deaths from ADEs (United States Drug Enforcement Administration, 2019). Tapentadol, with its dual mechanism of action acting as both a μ-opioid receptor agonist and noradrenaline reuptake inhibitor, has better tolerability than other commonly prescribed opioids due to its low μ-load (Romualdi et al., 2019). It was, therefore, unexpected to see its over-representation (10.3) in the third period. Furthermore, hydromorphone’s pronounced decrease in distribution in the past decade, in addition to its high potential for fatality and overdose rates, may contribute to its over-representation since the second period (+257.7%) (Lowe et al., 2017; Eidbo, et al., 2022). Methadone’s potential for overdose death (Kaufman et al., 2023) was overridden by its substantial distribution, explaining the under-representation (Furst et al., 2022). There has been some prior confusion regarding the safety of methadone relative to its role as the most distributed opioid by MMEs in the US (Piper et al., 2018). A prior report claimed that methadone accounted for less than 5% of opioid prescriptions dispensed but accounted for a third of opioid-related deaths (Webster et al., 2011). The data source (IMS Health, now known as IQVIA), however, did not include methadone from predominant sources of distribution from narcotic treatment programs and other federal programs (United States Drug Enforcement Administration, 2022).

The Drug Abuse Warning Network (DAWN), a nationwide public health surveillance system administered by the Substance Abuse and Mental Health Services Administration to monitor drug-related visits to hospital emergency departments, reported in its preliminary findings from its drug-related ED visits in 2021 that opioids were one of the top five substances for ED visits, with most reports being heroin-related, other opioids (oxycodone, buprenorphine, codeine, etc.), and fentanyl-related opioids (Substance Abuse and Mental Health Services Administration, 2022). Our findings indicate that, in the most recent period (2017–21), fentanyl’s relative distribution in adverse events and counts has decreased compared to some of the other opioids, highlighting that opioids like oxycodone, morphine, and hydromorphone might be contributing more to opioid adverse events compared to fentanyl. Combined with the reduced distribution based on the ARCOS reports regarding fentanyl counts in recent periods relative to other opioids, the adverse event-to-distribution ratio has decreased throughout the three periods. Like the FAERS database, it is important to note that the DAWN reports of opioids come from both mono and combo products (i.e., acetaminophen/oxycodone).

The main strengths of our novel study include the analysis of 11 commonly prescribed opioids, separated into uses for pain and OUD, with a new approach using both the FAERS and ARCOS database to quantify adverse events relative to the distribution of the opioids. The limitations to our study involve ARCOS and FAERS databases. A main limitation is that the ARCOS and FAERS do not provide formulation-specific information. There are no available data that break down the formulations of buprenorphine (i.e., by the route of administration). Additionally, the ARCOS does not filter out veterinary uses, although they were modest (Piper et al., 2020). The FAERS database might have over-represented the selected opioids because of duplicates, incomplete results, non-verifiable data, and uncertainty in adverse effect causalities (Veronin et al., 2019). In this case, most of the opioid ADE reports were from patients, with one-third being from medical professionals, which may contribute to the heterogenous quality of reports because of differing report behaviors between healthcare professionals and customers (Toki and Ono, 2020). The FAERS database is specifically populated by both mandatory (manufacturers of drugs) and voluntary (healthcare professionals, consumers, family members, etc.) adverse event reports. The FDA also raises cautions against making true conclusions from an analysis of the FAERS data (U.S. Food and Drug Administration, 2021). However, the FAERS database is a unique resource and has been used extensively by researchers for exploratory analyses and to identify hypotheses for further investigation (Sakaeda et al., 2013; Fang et al., 2014). The database has also been the primary surveillance database used to identify safety issues and adverse events or post-marketed drugs for decades as there is no other database that provides data on the relation of the drugs and ADEs (Wykowski and Swartz, 2005).

We assume that it is justified that individual opioids are equally likely to be reported to the FAERS and that it is the best database to date to analyze adverse events of prescription drugs, although future studies are needed to evaluate these hypotheses. However, as over three-quarters of FAERS submissions were completed by non-healthcare providers for oxycodone, hydrocodone, oxymorphone, and tapentadol, it is also possible that the US public is increasingly aware of the adverse effects of opioids (Macy, 2018) and is increasingly willing to utilize the FAERS to play their role in combatting the opioid epidemic. Further investigations with the FAERS and other similar databases will be necessary to determine if Figures 1, 3 are more informative.

Although the FAERS database has known limitations (US Food and Drug Administration, 2022), our exploratory analysis of the individual opioids provides novel findings that may guide further research in databases like the DAWN as the system only reports fentanyl-related and heroin-related products as separate groups while grouping other known opioids (Substance Abuse and Mental Health Services Administration, 2022). Another key limitation is that to date, there is no known database that consistently and accurately reports adverse events, abuse, and deaths. The CDC reporting of overdoses, for instance, has and continues to lack transparency with errors in counting overdose deaths (Peppin and Coleman, 2021). Another study reports that the death determination process is not uniform across the states (Kaufman et al., 2021).

Our analysis on FAERS and ARCOS databases demonstrated general increases in adverse events relative to opioid counts for the selected opioids, with varied relative individual adverse events when accounting for their distribution. It also provides a novel finding on individual opioids using both databases, further promoting research with other public databases like the Drug Abuse Warning Network. Emergency room visits in 2021 involving fentanyl (presumably predominantly illicit) were only one-fourth as common as other opioids (i.e., prescription), like oxycodone and hydrocodone (SAMHSA, 2022). Overall, the distribution pattern informs us of the need for continuous efforts to address the ADEs of specific opioids to inform healthcare policies and change the perspectives of healthcare providers on these drugs and their prescription practices.

## Data Availability

The original contributions presented in the study are included in the article/Supplementary Materials; further inquiries can be directed to the corresponding author.
