# Supplementary material for: Variation in adverse drug events of opioids in the United States
Source: Front Pharmacol. 2023 Mar 24;14:1163976. doi: 10.3389/fphar.2023.1163976 (PMC10079914; doi:10.3389/fphar.2023.1163976)
Supplement: Supplementary file 1 [file DataSheet1.docx]

**Supplementary Figure 1**. Percentage of reports of eleven opioids reported by healthcare professionals through the US Food and Drug Administration’s Adverse Events Reporting System from 2006-2021.


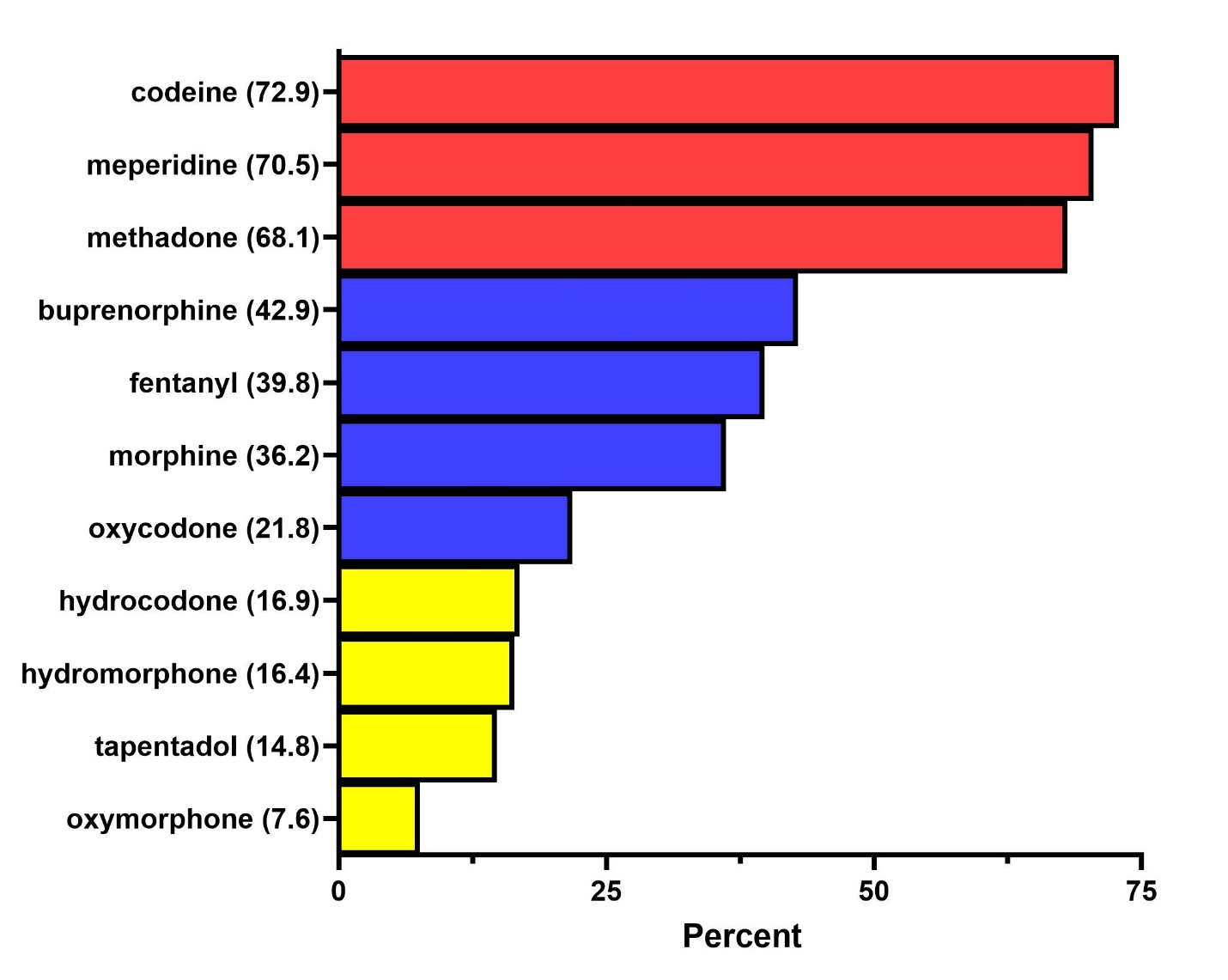


**Supplementary Table 1**. Opioid and search terms used in the US Food and Drug Administration’s Adverse Events Reporting System database.

____________________________________________________________________________

| Opioid | FAERS Search terms |
| --- | --- |
| Buprenorphine | “Buprenorphine”, “Buprenorphine Hydrochloride”, |
| Codeine | “Codeine”, “Codeine Camphorsulfonate”, “Codeine Hydrochloride”, “Codeine Phosphate” |
| Fentanyl | “Fentanyl”, “Fentanyl Citrate”, “Fentanyl Hydrochloride” |
| Hydrocodone | “Hydrocodone”, “Hydrocodone Bitartrate”, “Hydrocodone Hydrochloride”, “Hydrocodone Polistirex” |
| Hydromorphone | “Hydromorphone”, “Hydromorphone Hydrochloride” |
| Meperidine | “Meperidine”, “Meperidine Hydrochloride” |
| Methadone | “Methadone”, “Methadone Hydrochloride” |
| Morphine | “Morphine”, “Morphine Acetate”, “Morphine Hydrochloride”, “Morphine Sulfate”, “Morphine Tartrate” |
| Oxycodone | “Oxycodone”, “Oxycodone Hydrochloride” |
| Oxymorphone | “Oxymorphone”, “Oxymorphone Hydrochloride” |
| Tapentadol | “Tapentadol”, “Tapentadol Hydrochloride” |

**______________________________________________________________________________**

**Supplementary Table 2.** Death reports and their relative percentages by “reaction” and “outcome” in the US Food and Drug Administration’s Adverse Effect Reporting System for eleven prescription opioids for 2006-2021. “Reaction” indicates an adverse event leading to death, while “outcome” indicates the drug causing a direct death.

______________________________________________________________________________

**Opioid Total # of Reports Deaths as “reaction” Deaths as “outcome”**

oxycodone 159,441 23,357 (14.6%) 53,891 (33.8%)

fentanyl 106,644 16,309 (15.3%) 38,979 (36.6%)

morphine 102,411 17,088 (16.7%) 37,426 (36.5%)

buprenorphine 80,685 12,634 (15.7%) 22,861 (28.3%)

hydromorphone 64,454 15,064 (23.4%) 27,243 (42.3%)

hydrocodone 44,204 12,990 (29.4%) 24,677 (55.8%)

oxymorphone 31,154 12,654 (40.6%) 22,098 (70.9%)

tapentadol 29,290 11,579 (39.5%) 20,020 (68.4%)

methadone 27,454 2,628 (9.6%) 13,051 (47.5%)

codeine 16,731 705 (4.2%) 4,691 (28.0%)

meperidine 5,501 75 (1.4%) 406 (7.4%)

**______________________________________________________________________________**
